# The Effect of Topical Ocular Anesthetic Proparacaine on Conjunctival and Nasal Mucosal Flora in Dry Eye Disease Patients

**DOI:** 10.3390/jcm7040073

**Published:** 2018-04-09

**Authors:** Ozlem Onerci Celebi, Ali Riza Cenk Celebi

**Affiliations:** 1 Eregli State Hospital Department of Otorhinolaryngology, TorosMah. Yurt Cad. No. 13, 42000 Eregli, Konya, Turkey; oonerci@yahoo.com; 2 Acibadem University School of Medicine, Atakent Education and Research Hospital Department of Ophthalmology, TurgutOzalBlvrd. No.16, 34303 Kucukcekmece, Istanbul, Turkey

**Keywords:** anesthetic, antibacterial, conjunctival flora, nasal mucosal flora, dry eye, proparacaine

## Abstract

The aim of this study was to investigate the effect of topically applied ocular anesthetic proparacaine on conjunctival and nasal bacterial mucosal flora in patients with dry eye disease. A Schirmer test was done with (group 1) and without (group 2) topical anesthetic proparacaine to 40 patients in each group. Conjunctival and nasal cultures were obtained before and 10 min after performing the Schirmer test. The bacterial culture results and the isolated bacteria were recorded in two groups. Patients’ mean age was 62 years (70 female, 10 male). Before the application of topical anesthetic, 50 (62.5%) and 62 (77.5%) had positive conjunctival and nasal culture, respectively, with the most commonly isolated organism being coagulase-negative *Staphylococcus* in each group. In group 1 the conjunctival bacterial culture positivity rate decreased from 26 (65%) to six (15%) eyes (*p* < 0.001); however, this rate decreased slightly from 24 (60%) to 20 (50%) eyes in group 2 (*p* > 0.05). For the nasal cultures, the bacterial culture positivity rate decreased from 80% to 20% and from 75% to 65% in groups 1 (*p* < 0.001) and 2 (*p* > 0.05), respectively. Topical ocular anesthetic proparacaine has antibacterial activity in both conjunctival and nasal flora in patients with dry eye disease.

## 1. Introduction

In vivo non-ophthalmological trials and more recently in vitro and in vivo ophthalmological studies have provided consistent evidence demonstrating the antibacterial activity of topical anesthetics [1,2,3]. There is agreement across different studies that most pathogens may be affected by certain types of anesthetics [4,5]. The antibacterial activity of proparacaine, a common topical anesthetic agent, has previously been demonstrated against ocular bacterial flora [6,7].

The ocular surface of healthy individuals inherently supports a small population of bacteria, typically coagulase-negative staphylococci (CNS), which are believed to exist as commensals on the mucosa [8]. The production of lipases and toxins by many of these colonizing bacteria may induce ocular surface cellular damage and destabilization of the lipid layer of the tear film, contributing to tear film instability, inflammation, and symptoms of significant ocular irritation [9]. Similar symptoms commonly occur in dry eye, without evidence of purulent exudative infection. Schirmer test is commonly used to evaluate and confirm dry eye disease. Schirmer test can be performed with or without topical anesthesia [10].

The nasal mucosa serves as a reservoir of organisms for the conjunctiva and the nasolacrimal system. A reduction of perioperative conjunctival bacterial flora was shown with nasal application of Mupirocin [11]. Additionally, the nasal mucosa is exposed to antibiotics after its topical application to the eye [12]. 

As the nasal cavity and the eye are related to each other by the nasolacrimal duct, infections in one structure carry the risk of transmitting the infection to the other [13]. This suggests that there could be a microbiological interaction between the nose and the eye. Although the anatomical relationship between the ocular surface and the nasal cavity is well known, studies regarding the effects of various chemicals applied to the ocular surface on the nose are limited. 

The aim of the study was to investigate the effect of topically applied ocular anesthetic proparacaine on conjunctival and nasal bacterial mucosal flora.

## 2. Experimental Section

This study was carried out with the approval of the Local Ethical Committee. In accordance with the tenets set forth in the Declaration of Helsinki for research involving human subjects, written informed consent was obtained from all subjects prior to their participation in the study.

The study included 80 dry eye disease patients who were on treatment with preservative-free topical ocular lubricant therapy five times a day (0.15% sodium Hyaluronic Acid, EyestilUnidose, SIFI S.p.A). Forty of the patients had dry eye disease diagnosed by (but not limited to) the Schirmer test with anesthetics (group 1). A commercially available solution of 0.5% proparacaine (Alcaine 0.5%, Alcon-Couvreur, B-2870 Puurs, Belgium) was used for ocular anesthesia during testing. For the remaining 40 patients, dry eye was diagnosed using (but not limited to) the Schirmer test without anesthetics (group 2). During routine follow-up examination of the patients, the same Schirmer test method was used for consistency. Patients with active ocular infection, eyelid deformities, history of use of any antibiotics in the last three months, and history of any ocular surgeries were excluded. All patients had patent passage of nasolacrimal system confirmed by nasolacrimal irrigation.

Before performing the Schirmer test, an initial conjunctival culture was obtained from the inferotemporal conjunctival sacs of both eyes of the patients. In the meantime, two sterile cotton-tipped applicators moistened with soy broth were placed in the right and left nostril (one each) and rolled gently. Next the Schirmer test was performed. For group 1, one drop of proparacaine eyedrop was applied. Afterwards, the test strip paper was placed on the lateral inner portion of the palpebral conjunctiva for 5 min. After taking out the test strips, we waited another 10 min for the patients’ cooperation for another round of obtaining conjunctival and nasal cultures with the same methods described above. For all phases, the conjunctival and nasal cultures were immediately plated onto blood agar and chocolate agar culture medium. The procedures used for the culturing and identification were consistent with current laboratory methods and culture media were defined as positive if organisms were grown along the line of inoculation site and/or one or more colony was present on the agar plates. The first inspection of the cultures was carried out to detect bacteria 24–72 h after incubation. The bacterial culture results and the isolated bacteria were recorded both prior to and after the Schirmer test.

### Statistical Evaluation

Statistical analyses were performed using SPSS for Windows (ver. 22.0; SPSS Inc., Chicago, IL, USA). Descriptive statistics including mean age of the study population and culture positivity rates were calculated. The Mann-Whitney U test was used to evaluate the statistical significance of the results by comparing the culture positivity rates between groups 1 and 2. The Wilcoxon signed rank test was used to compare the culture positivity rates prior to and after the Schirmer test for each group. A *p* value of less than 0.05 was considered significant.

## 3. Results

We obtained smears of the conjunctival sacs and nostrils of 80 patients prior to the Schirmer test. The patient population was comprised of 70 women and 10 men with a mean age of 62 years. Of the 80 patients, 50 had positive conjunctival culture and 62 had positive nasal culture for at least one type of bacteria. Prior to the Schirmer test in groups 1 and 2, 26 (65%) and 24 (60%) patients yielded positive conjunctival cultures, respectively. This difference in the conjunctival bacterial culture positivity rate was not found to be statistically significant (*p* = 0.123, Mann-Whitney *U* test). The bacterial culture positivity rates were higher in nasal cultures, which were 80% (32/40) and 75% (30/40) in groups 1 and 2, respectively. The difference of this nasal bacterial culture positivity rate was not statistically significant between groups 1 and 2 (*p* = 0.132, Mann-Whitney *U* test). The types of bacterial species isolated were mostly homogenous between groups 1 and 2 for both conjunctival and nasal cultures, with the most commonly isolated organism being coagulase-negative staphylococci. The distribution of cultured organisms prior to Schirmer test in groups 1 and 2 for both conjunctival and nasal cultures is summarized in Table 1.

Ten minutes after performing the Schirmer test, six (15%) and 20 patients (50%) had conjunctival culture positivity in groups 1 and 2, respectively. This difference in the conjunctival culture positivity rate was found to be statistically significant between groups 1 and 2 (*p* < 0.001, Mann–Whitney *U* test). The bacterial culture positivity rates in nasal cultures were 20% (8/40) and 65% (26/40) in groups 1 and 2, respectively. The difference in this nasal bacterial culture positivity rate 10 min after performing the Schirmer test between groups 1 and 2 was found to be statistically significant (*p* < 0.001, Mann-Whitney *U* test). The most commonly isolated organism was coagulase negative staphylococci 10 min after the Schirmer test. The distribution of cultured organisms in groups 1 and 2 10 min after the Schirmer test for both conjunctival and nasal cultures is summarized in Table 2.

The conjunctival bacterial culture positivity rate decreased from 65% (26/40) to 15% (6/40) in group 1. This decrease was found to be statistically significant (*p* < 0.001, Wilcoxon signed rank test). There was also a decrease in group 2. The rate decreased from 60% (24/40) to 50% (20/40) but this decrease was not found to be statistically significant (*p* = 0.084, Wilcoxon signed rank test).

The nasal bacterial culture positivity rate decreased from 80% (32/40) to 20% (8/40) in group 1. This decrease was found to be statistically significant (*p* < 0.001, Wilcoxon signed rank test). There was also a decrease found in group 2. The rate decreased from 75% (30/40) to 65% (26/40), but this decrease was not found to be statistically significant (*p* = 0.065, Wilcoxon signed rank test).

## 4. Discussion

In this prospective study, we examined the effects of the topically applied ocular anesthetic proparacaine on the nasal cavity flora and found that the nasal cavity flora was affected by proparacaine in patients diagnosed with dry eye syndrome. In other words, one of the most frequently used commercially available topical anesthetic proparacaine has a substantial antibacterial effect on pathogens commonly isolated from nasal bacterial flora in addition to the conjunctival bacterial flora in dry eye disease patients.

As the nasal cavity and the eye are related to each other by the nasolacrimal duct, along with their adjacency, infections in one structure carry the risk of being able to infect the other [13]. Similar bacteriological flora could be present between the nose and the eye, which are structures with anatomic intertwining. Coagulase negative *Staphylococcus*, *S. aureus*, and *Corynebacterium* are the most common ocular microorganisms isolated from the conjunctiva and the nasal cavity [14]. This distribution could also support this anatomic intertwining. In our study the most commonly isolated microorganisms from the conjunctiva and nasal cavity were coagulase negative *Staphylococcus*, *S. aureus* and *Corynebacterium*, which was in accordance with the literature.

In a previous study, Mullin and associates [6] demonstrated the bacteriostatic and bactericidal effects of topically applied proparacaine. They observed that proparacaine strongly inhibited the growth of one of the common commensal *S. epidermidis* at a 0.5% concentration. In our current study it was also demonstrated that a concentration of 0.5% of proparacaine, which is commonly used during ophthalmology practice, has an antibacterial effect on both conjunctival and nasal flora. 

The antibacterial effect of topical anesthetics could be related to the changes in lysozymes and lysozomal enzymes, which are important antibacterial components of tear film. The enzyme activity of lysozyme per fluid volume in tears obtained from anesthetized eyes was found to be higher than the ones obtained from non-anesthetized eyes [15]. 

*S. epidermidis* found in the conjunctival sac was observed as a major component in the nasal cavity as well [14]. In the current study, coagulase-negative *Staphylococcus*, *Corynebacterium* species, and *Staphylococcus aureus* were the bacteria most commonly isolated from the nasal cavity. Previous studies have supported our results [14,16]. 

The main source of infection in ophthalmology is usually the patient’s endogenous bacterial flora [17]. It is worth noting that cataract surgery is the most commonly performed operation in ophthalmology and the conjunctival bacterial flora can enter the anterior chamber from the conjunctival cul de sac during operation or even after the first incision [18]. Ophthalmic surgeons usually begin the operation shortly after the administration of a topical anesthetic. Therefore, the decrease in bacterial load of the conjunctiva with topical anesthesia can help decrease the rate of endophthalmitis. 

One important aspect of topical anesthetic should be kept in mind. It has been reported in a previous non-ophthalmological study that anesthetic agents had the potential for low yield of bacterial culture due to their antibacterial activity [19]. Thus, this antibacterial activity of topical anesthetics can partially explain the negative cultures in some bacterial keratitis and corneal ulcer cases [3].

It is reported that the culture positivity rate of ocular bacteria increases with age [20]. Age-related changes include a decrease in resistance and immune function, decreased lacrimation, and impaired self-cleansing of the ocular surface due to narrowing of the nasolacrimal duct. All of these contribute to an increased rate of bacterial culture positivity. In our elderly population, the culture positivity rate was found to be higher than in the literature [14]. 

Reducing or changing the distribution of the normal nasal commensal bacterial flora can increase the frequency of pathogenic nasal Methicilline-Resistant *Staphylococcus aureus* (MRSA) strains, which is a common cause of nasocomial infections in a hospital setting [21,22]. Human studies have confirmed that nasal commensal bacteria such as *S. epidermidis* compete with *S. aureus* for the same niche [22], so reducing the number of colonized commensal *S. epidermidis* in nasal flora with any antibacterial agents like proparacaine may increase the nasal MRSA colonization. One recent study also showed that the normal nasal flora bacteria *S. epidermidis* significantly suppresses the infectivity of various influenza viruses, so reducing the nasal *S. epidermidis* colonies could result in influenza outbreaks [23]. 

Our study has some limitations. It should be noted that the sample size was not too large. The prospective design of the study can help us assess the antibacterial effect of proparacaine on nasal mucosal flora after short-term use; however, a long-term follow-up study would be needed to see whether this effect is persistent or not. The other limitation is related to the need for a vehicle control group; we did not compare our results with another group that used vehicle eye drops as a control. So it would be better to verify our results with an appropriate vehicle control. One other limitation of the study is related to the ingredient benzalkonium chloride 0.01% (BAC), which is present in commercially used topical proparacaine. The efficacy of BAC as a disinfectant is well known [24] and the antibacterial effect of commercially available proparacaine may be related to the effect of BAC. Another limitation of the study could be related to the study population, which was comprised of dry eye disease patients. There could be some difference between mild and severe dry eye diseases or the effect of proparacaine on conjunctival flora may be different in healthy populations.

## 5. Conclusions

This is the first study to show the antibacterial activity of topically applied proparacaine on nasal flora in patients with dry eye disease. Additional clinical and laboratory studies with larger cohorts are needed in order to understand the antibacterial effects of topically applied proparacaine and verify our preliminary results.

## Figures and Tables

**Table 1:** The number of cultured organisms prior to Schirmer test in groups 1 and 2 for both conjunctival and nasal cultures.

Microorganism Type	Group 1 * (40 Patients)		Group 2 ** (40 Patients)	
	Conjunctival Culture	Nasal Culture	Conjunctival Culture	Nasal Culture
Coagulase-negative *Staphylococcus*	40% (16)	40% (16)	35% (14)	35% (14)
*Staphylococcus aureus*	15% (6)	20% (8)	15% (6)	20% (8)
*Corynebacterium* spp.	5% (2)	15% (6)	5% (2)	15% (6)
*Streptococcus viridans*	5% (2)	5% (2)	5% (2)	5% (2)

* Patients whose dry eye was diagnosed using the Schirmer test with anesthetics, ** Patients whose dry eye was diagnosed using the Schirmer test without anesthetics.

**Table 2:** The number of cultured organisms 10 min after performing the Schirmer test in groups 1 and 2 for both conjunctival and nasal cultures.

Microorganism Type	Group 1 * (40 Patients)		Group 2 ** (40 Patients)	
	Conjunctival Culture	Nasal Culture	Conjunctival Culture	Nasal Culture
Coagulase-negative *Staphylococcus*	10% (4)	10% (4)	12	12
*Staphylococcus aureus*	5% (2)	5% (2)	15% (6)	17.5% (7)
*Corynebacterium* spp.	0% (0)	5% (2)	2.5% (1)	15% (6)
*Streptococcus viridans*	0% (0)	0% (0)	2.5% (1)	2.5% (1)

* Patients whose dry eye was diagnosed using the Schirmer test with anesthetics; ** Patients whose dry eye was diagnosed using the Schirmer test without anesthetics.
